# Plant cell walls as a key driver of plant–pathogen coevolution

**DOI:** 10.1093/femsre/fuag023

**Published:** 2026-05-15

**Authors:** Cristian Carrasco-López, Sergio López -Cobos, Andrea Sánchez-Vallet

**Affiliations:** Centro de Biotecnología y Genómica de Plantas, Universidad Politécnica de Madrid (UPM) – Instituto Nacional de Investigación y Tecnología Agraria y Alimentaria/Consejo Superior de Investigaciones Científicas (INIA/CSIC), Campus de Montegancedo UPM, 28223 Pozuelo de Alarcón, Madrid, Spain; Centro de Biotecnología y Genómica de Plantas, Universidad Politécnica de Madrid (UPM) – Instituto Nacional de Investigación y Tecnología Agraria y Alimentaria/Consejo Superior de Investigaciones Científicas (INIA/CSIC), Campus de Montegancedo UPM, 28223 Pozuelo de Alarcón, Madrid, Spain; Departamento de Biotecnología-Biología Vegetal, Escuela Técnica Superior de Ingeniería Agronómica, Alimentaría y de Biosistemas, UPM, 28040 Madrid, Spain; Centro de Biotecnología y Genómica de Plantas, Universidad Politécnica de Madrid (UPM) – Instituto Nacional de Investigación y Tecnología Agraria y Alimentaria/Consejo Superior de Investigaciones Científicas (INIA/CSIC), Campus de Montegancedo UPM, 28223 Pozuelo de Alarcón, Madrid, Spain

**Keywords:** plant cell wall modifying enzymes, cell wall-derived elicitor, plant resistance, molecular arms race, cell wall integrity, effector

## Abstract

Plant cell walls constitute a central battleground in the molecular arms race, shaping plant–pathogen interactions. Pathogens deploy hydrolytic enzymes to breach plant cell walls, whereas plants perceive cell wall damage through sophisticated resistance mechanisms. Coevolutionary dynamics between both organisms continue, as pathogens evolve strategies to evade host recognition while plants counteract the activity of hydrolytic enzymes. Consequently, in this interorganismal interaction, plant cell walls emerge as critical for the outcome of the disease. In this review, we expand the classical arms race model and incorporate the contribution of plant cell walls in plant immunity and filamentous pathogen virulence.

## Teaser

In this review, we explore the contribution of plant cell walls in plant–pathogen coevolution, highlighting how filamentous pathogens breach this barrier to access nutrients and colonise the host, and how plants sense cell wall breakdown and induce an immune response.

## Introduction

Pathogens are engaged in a molecular arms race with their hosts in which plants evolve to restrict the progression of the pathogen, while pathogens evolve to circumvent the immune response triggered by the host. This model has been widely described in the context of the so-called effectors and resistance proteins (Jones and Dangl 2006, Dodds and Rathjen 2010). Effectors are pathogen-secreted molecules that promote plant infection. Plants use these same molecules as signals to detect pathogen invasion. Through plant resistance proteins (R), they recognize certain forms of effectors and trigger an immune response that hinders pathogen progression. This imposes a strong evolutionary pressure on the pathogen to escape host recognition. Pathogens can evade recognition primarily by altering effector sequences or by deploying additional effectors that suppress host immune responses (Sánchez-Vallet et al. 2018). Recognition can occur either through the direct binding of the effector and the resistance protein or indirectly, through the recognition of the outcome of effector activity. The latter mechanism, described in the guard model (Bent and Mackey 2007), frequently involves recognition of proteins targeted by effectors (Van Der Hoorn and Kamoun 2008). Yet, effectors not only target proteins, but they also act on molecules of distinct nature, such as carbohydrates from plant cell walls (Molina et al. 2024b, Meile et al. 2025). However, nonp roteinaceous targets have been largely overlooked in the classical evolutionary molecular arms race model.

Plant cell walls constitute a physical barrier and a source of nutrients for the pathogen. During plant infection, pathogens target cell wall components to colonize the host (Hématy et al. 2009, Munzert and Engelsdorf 2025). Hydrolysis of cell wall polysaccharides is largely mediated by the secretion of a particular type of effectors harbouring catalytic domains and classified as cell wall modifying enzymes (CWMEs). These CWMEs are specialized to modify and hydrolyze each plant cell wall component at specific stages of the infection (Bradley et al. 2022). Although crucial for infection, the activity of CWMEs alters cell wall structure and composition and leads to the release of oligosaccharides, which can be sensed by plants as danger signals known as Damage-Associated Molecular Patterns (DAMPs), and lead to the activation of defence responses (Molina et al. 2024a, Bacete et al. 2018, Dünser et al. 2019, Wolf 2022). The dual role of CWMEs creates a strong evolutionary constraint for pathogens: they should degrade plant walls to obtain nutrients and breach the wall, while minimizing DAMP release and recognition. This conflict has led to an ongoing evolutionary arms race at the plant cell walls, driving pathogens to fine-tune enzyme release and activity, while hosts develop sophisticated detection mechanisms for CWMEs and their products.

This review examines the strategies by which filamentous pathogens interact with plant cell walls to enhance virulence while evading host immune activation. Previous reviews have addressed plant cell wall-derived DAMPs and plant immunity (Molina et al. 2024a, Sun et al. 2025b, Bacete et al. 2018, Bacete and Hamann 2020, Sánchez-Vallet et al. 2020, De Lorenzo and Cervone 2022, Wolf 2022, Pinto et al. 2025), as well as the structural and compositional features of plant cell walls (Cosgrove 2024, Fuertes-Rabanal et al. 2025). Building on these foundations, we highlight recent advances that contribute to our understanding of the coe volution of plants and filamentous pathogens at the plant cell wall.

## The plant cell wall: structure and composition

Cell walls and cuticles are one of the first contacts for pathogens during plant infection. Cell walls are dynamic structures that protect plant cells and adapt to environmental conditions (Cosgrove 2024, Fuertes-Rabanal et al. 2025). They are formed primarily by carbohydrates and phenolic compounds, with lower amounts of proteins. Some specialized cells deposit an additional secondary cell wall between the plasma membrane and the primary cell wall. These secondary cell walls have a higher content of cellulose and lignin and provide structural support (Burton et al. 2010). Lignin is a phenolic polymer that contributes to cellular tensile strength and serves as a pathogen barrier preventing penetration into plant tissues (Boerjan et al. 2003, Miedes et al. 2014). Suberin is a lipid polyester deposited in specialized cells; it limits water movement and is located in plant protective layers, including bark of trees, abscission zones, in root endodermis and exodermis, in the periderm, seed coat, and in the bundle sheath cells (Philippe et al. 2020, Serra and Geldner 2022). Epidermal cells are covered by cuticles that coat the aerial parts of plants. These are formed by cuticular waxes and cutin, and prevent diffusion of water, gases, and solutes (Schreiber 2010, Philippe et al. 2020). Although this external polymeric matrix is usually not considered a plant cell wall component, it remains physically connected to the epidermal wall and constitutes a major defensive barrier against pathogen invasion, which has driven the evolution of diverse strategies in the pathogen to breach it (Yeats and Rose 2013).

A network of cellulose microfibrils provides resistance to deformation in cell walls. This network of microfibrils is tethered by hemicelluloses and embedded in a matrix of pectin (Cosgrove 2024). Cellulose is a polymer of β-1,4-d-glucose that can reach a degree of polymerization (DP) of about 10 000 glucose units. The association of several cellulose chains, which are stabilized by hydrogen bonds and Van der Waals forces, forms cellulose microfibrils, which provide high tensile strength. Cellulose is the main component of plant cell walls in all plant species (Cosgrove 2024). Hemicelluloses, including xyloglucans, xylans, mannans, and β-1,3/1,4-mixed-linked glucans (MLGs), are diverse in composition and structure and bind to cellulose (Burton et al. 2010, Scheller and Ulvskov 2010). In type I primary cell walls from dicots and noncommelinoid monocots, the most abundant hemicellulose is xyloglucans, but other hemicelluloses such as xylans and mannans are also typical. In contrast, in type II cell walls from grasses, xylans are the most abundant hemicelluloses (Carpita and Gibeaut 1993, Scheller and Ulvskov 2010). Depending on the substitutions of the xylan chain, three types of xylans can be distinguished: glucuronoxylans, glucuronoarabinoxylans, and arabinoxylans (Tryfona et al. 2023). Arabinoxylan substitutions with phenolic acids (ferulic acid and p-coumaric) are key characteristics of type II primary cell walls. Mannans are the most widely distributed hemicelluloses and are present in all plant species (Voiniciuc 2022). They are formed by a β-1,4-mannan backbone (homomannan) or a β-1,4-linked mannan–glucose backbone (glucomannan), in which the mannose residues can be substituted with galactosyl side chains (galactoglucomannan). MLGs are hemicelluloses exclusively present in grasses and some lower plant cell walls (Scheller and Ulvskov 2010, Voiniciuc 2022).

Pectins are acidic polysaccharides and play major roles in plant development and defence. They are important for the viscoelastic properties of the cell walls and are the main component in the middle lamella (Cosgrove 2024). They are highly diverse and complex polysaccharides, and therefore, their structural conformation remains largely unknown. Pectins contribute to the porosity and adhesion of cell walls. They include several structural classes, including homogalacturonan, xylogalacturonan, apiogalacturonan, and rhamnogalacturonan, and they are primarily composed of α-1,4-d-galacturonic acid residues (Ishii 1997, Ridley et al. 2001, Mohnen 2008, Shi et al. 2017, Cosgrove 2024).

In addition to carbohydrates, plant cell walls harbour proteins with distinct functions. Some proteins are bound to the cell wall, such as extensins and hydroxyproline-rich proteins. Other proteins include enzymes, such as endoglucanases and pectinases, or are hydroxyproline-rich glycoproteins, including lectins and arabinogalactan proteins.

The general components of cell walls are conserved in plants. However, the specific composition differs largely, depending on the species, the cell type, and the developmental stage. For instance, cell walls from dicotyledonous plants are rich in pectins and xyloglucans, while *Poaceae* have relatively small amounts of pectin and high amounts of xylans and MLGs (Burton et al. 2010). Walls from xylem cells are thicker and have a high content of lignin, and young cells have more porous walls that enable diffusion of water and nutrients between cells (Burton et al. 2010). Thus, pathogen infection requires a specialized and highly sophisticated repertoire of CWMEs to colonize specific host species and organs.

## Pathogen attack: breaking the cell wall

**Figure 1:**
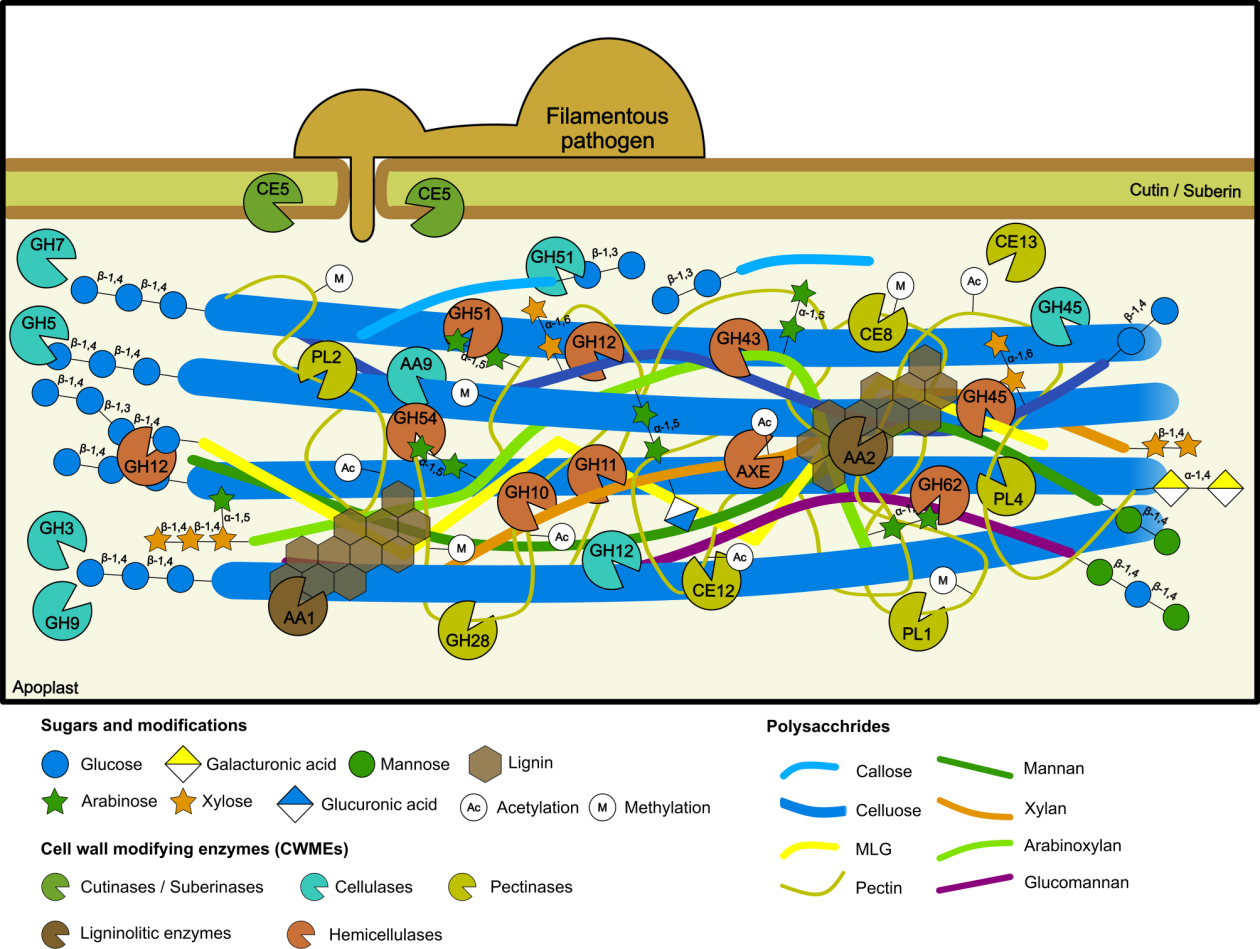
Pathogen strategies to breach plant cell walls. During plant infection, filamentous pathogens secrete specialized cell wall modifying enzymes (cutinases, suberinases, cellulases, pectinases, hemicellulases and ligninolitic enzymes) to modify plant cell walls and colonize the host.

Filamentous pathogens breach plant cell walls by secreting a large repertoire of hydrolytic enzymes. Carbohydrate-Active enZymes (CAZymes) modify and cleave cell wall carbohydrates, and are classified into six main classes depending on their catalytic activities (Kubicek et al. 2014). Glycoside hydrolases (GHs) catalyse the breakdown of glycosidic linkages; carbohydrate esterases (CEs) the de-O or de-*N*-acylation of substituted saccharides; polysaccharide lyases (PLs) the cleavage of uronic acid-containing polysaccharides; and auxiliary activities (AAs), such as lytic polysaccharide monooxygenases (LPMOs), facilitate the access of other CAZymes to their substrates (Drula et al. 2022). The two remaining classes are not primarily involved on the breakage of glycans. Glycosyltransferases catalyse the transfer of monosaccharide donors to carbohydrates, achieving structural changes in plant cell wall glycans that can alter virulence, and carbohydrate-binding modules (CBMs) are sugar-binding proteins without catalytic domains (Drula et al. 2022). Plant cell wall polymers are not spatially isolated and, therefore, decomposition of cell walls during plant infection presumably requires the synergistic and concerted action of multiple enzymatic activities (Fig. 1).

### Cutin and suberin degradation

Pathogens have evolved multiple invasion strategies to overcome the cuticle, including entry through open stomata or wounds, as well as direct penetration using specialized structures such as appressoria and the release of cutinases (Mendgen et al. 1996). Cutinases are expressed early during infection in several plant pathogens when they are in close contact with the cuticle (Mendgen et al. 1996). Cutinases mediate virulence by degrading the cuticle as a physical barrier. For example, expression of cutinases from *Fusarium solani* f. sp. *pisi* in the opportunistic pathogen *Mycosphaerella* sp. increases its penetration and colonisation rates in papaya plants (Dickman et al. 1989). In *Magnaporthe grisea*, *Alternaria alternata*, and *Colletotrichum gloesporiodes*, cutinases are required for infection of intact plants, but not of wounded plants (Dickman and Patil 1986, Skamnioti and Gurr 2007, Ma et al. 2019), highlighting the critical role of breaking the cuticle for penetration of intact plants. As one of the first contacts with plants, cutin provides virulence signals for several plant pathogens. *Maagnaporthe grisea*, *Magnaporthe oryzae* and *Ustilago maydis* detect cutin monomers and hydrophobic surface, triggering the development of appressoria (Skamnioti and Gurr 2007, Mendoza-Mendoza et al. 2009, Lanver et al. 2014). In *U. maydis*, cutin monomers control the expression of genes encoding pectinases, cellulases and arabinofuranosidases that potentially promote penetration through the plant cell wall (Lanver et al. 2014). In *M. grisea*, the aberrant appressoria formed by cutinase mutants are restored by supplementation with cutin monomers (Skamnioti and Gurr 2007). These results highlight the relevance of cutin as both a defence barrier and a source of signals that initiate the infection machinery in plant pathogens.

Suberin is highly resistant to degradation, thereby preventing root colonization by pathogens and influencing the composition of the root microbiota (Fröschel et al. 2021, Salas-González et al. 2021, Shukla and Barberon 2021, Serra and Geldner 2022). Root-associated microbiota modulate endodermal lignification and suberization levels (Salas-González et al. 2021). The pathogenic fungus *Verticillium longisporum* degrades the endodermis to access the root central cylinder and enable pathogen colonisation (Fröschel et al. 2021). Other root-associated fungi produce hydrolytic enzymes, such as cutinase-like proteins, lipases, and oxidases that degrade suberin. For example, the CE5 cutinase *Fs*CUT1 from *F. solani* degrades both cuticle and suberin (Fernando et al. 1984). The *Verticillium dahliae* CE5 *Vd*CUT11 degrades suberin and is required for the infection of cotton roots (Gui et al. 2018).

### Cellulose degradation

Cellulose is highly recalcitrant to degradation by filamentous pathogens. LPMOs, which are monocopper enzymes, break crystalline cellulose through oxidative reactions. Consequently, LPMO proteins from the AA9 family increase the accessibility to cellulose of endoglucanases (GH12, GH45, or GH51), which cleave internal glycosidic bonds, and to exoglucanases (GH3, GH5, GH7, and GH9), leading to the release of d-glucan monosaccharides (Couturier et al. 2016). The contribution of AA9 to virulence remains to be fully understood (Li et al. 2020, Tamburrini et al. 2024). The *Colletotrichum orbiculare Co*AA9 is essential for appressorium formation, and its C-terminal disordered region promotes AA9 homodimerization, enhancing both cellulose substrate binding and degradation (Tamburrini et al. 2024). *Magnaporthe oryzae Mo*AA91 does not have cellulase activity on its own, but it exhibits a synergistic effect when combined with cellulases. Despite *Mo*AA91 role in appressorium formation, the contribution of *Mo*AA91 catalytic activity remains unclear, as mutations in the AA9 catalytic site did not affect appressorium development or virulence (Li et al. 2020). While LPMOs provide the initial disruption, endoglucanases and exoglucanases are also essential for the full breakdown of cellulose and virulence. Loss of the GH7 *Vd*GH7a in *V. dahliae* reduced tolerance to osmotic stress, impaired conidiation, and limited growth on β-1,4-glucans and penetration in cotton leaves (Lü et al. 2025). In the oomycete *Phytophthora sojae*, the glucanase *Ps*GH7d is required for virulence on soybean (Liu et al. 2025). The GH51 protein Erc1 from *U. maydis* mediates fungal movement in bundle sheath cells of maize. Biochemical assays demonstrated the capacity of Erc1 to bind to various plant cell wall polysaccharides, including cellulose; however, Erc1 has only been shown to have exo-β-1,3-glucanase activity. Because β-1,3 bonds are not common in structural glucans of plant cell walls, Erc1 may primarily degrade immunogenic fragments such as laminarihexaose, although its role in wall loosening cannot be excluded (Ökmen et al. 2022).

### Hemicellulose degradation

Xylan is composed of β-1,4-linked chains with side branches of mainly arabinan and mannose. Complete degradation of the xylan backbone requires debranching and xylanase activity. Thus, both endo- and exo-acting enzymes targeting side chains are crucial for hemicellulose decomposition (Biely and Tenkanen 1998). Xylan is an excellent target for pathogen CWMEs because structural xylans are absent from microbial cell walls and, thus, released xylanases do not damage their own cell wall. Pathogens secrete xylanases during infection. The oomycete *P. sojae* secretes *Ps*XEG1, a GH family 12 enzyme which possesses xyloglucanase and β-glucanase activities, and is essential for pathogenesis (Ma et al. 2015b). Similarly, the GH12 protein EG1 from *Fusarium sacchari* hydrolyses cellulose and xyloglucan. During sugarcane infection, EG1 contributes to necrosis by releasing cellulose-derived immunogenic fragments, and in this way, it contributes to virulence (Wu et al. 2026). In *Puccinia striiformis* f. sp. *tritici*, the most highly expressed xylanase is the GH10 *Pst*Xyn1. This enzyme degrades xylan in plant cell walls. Silencing of *Pst*Xyn1 impaired epiphytic hyphal growth and hindered the development of disease symptoms (Ma et al. 2024). The *Colletotrichum fructicola* and *Botrytis cinerea* GH11 proteins, *Cf*Xyn11A and *Bc*Xyn11A, are also required for virulence and xylan usage as a nutrient (Brito et al. 2006, Han et al. 2025). Xylan degradation is not restricted exclusively to pathogens. The mutualistic fungus *Serendipita indica* activates a broad xylan-degrading program during interactions with monocots. Among its enzymes, *Si*GH11 (an endoxylanase) and *Si*AXE (an esterase) act synergistically: *Si*GH11 cleaves the backbone while *Si*AXE deacetylates oligosaccharides. This dual activity is advantageous, as it prevents the induction of the host immune response triggered by xylan oligosaccharides, and the oligosaccharide-derived products are utilized by GH43 exoxylanases to release xylose and feed fungi (Brands et al. 2025).

Arabinoxylan is also absent from microbial cell walls and targeted by arabinofuranosidases from pathogens, including members of the GH43, GH51, GH54, and GH62 families (Drula et al. 2022). During infection of rice, *M. oryzae* secretes to the apoplastic space the arabinofuranosidase *Mo*AbfB. *Mo*AbfB degrades plant cell wall structures and contributes to *M. oryzae* virulence. However, *Mo*AbfB also contributes to the release of elicitors of resistance, and the protein activates a plant immune response (Wu et al. 2016).

Together with xylans and arabinans, β-mannans are a major hemicellulose component targeted by pathogens. β-mannanases are widespread in plant filamentous pathogens, but direct genetic evidence for individual β-mannanases acting as virulence determinants is still missing. Hemicelluloses of type II cell walls, such as in *Poaceae* or horsetail, contain MLGs. These polysaccharides are susceptible to the digestion by endoglucanases and lichenases, an MLG-specific endoglucanase which cleaves β-1,4 bonds next to the β-1,3 bonds (Fry et al. 2008). Pathogenic MLG-degrading enzymes are produced by *M. oryzae* (*Mo*Cel12A/B) and *Zymoseptoria tritici* (*Zt*GH45). Both pathogens express these endoglucanases during infection. However, their catalytic activity releases highly immunogenic MLG-derived fragments that hinder pathogen virulence (Yang et al. 2021, Rebaque et al. 2025).

### Pectin degradation

Pectin breakdown is achieved by the activity of GH28 polygalacturonases (PGs) and rhamnogalacturonases, together with PL1 and PL2 pectate lyases and PL4 rhamnogalacturonan lyases. PGs cleave the glycosidic bond by hydrolysis, while pectin and pectate lyases by β-elimination. Since pectin is often methylesterified and/or acetylesterified, the activity of CE8 methyl-esterases, and CE12 and CE13 acetyl-esterases is probably required before backbone-cleaving enzymes can act (Kubicek et al. 2014, Kontogiorgos 2020) during plant infection.

*Botrytis cinerea* pectinolytic enzymes include several PGs, pectin methyl esterases (PMEs), pectin lyases (PNLs), and two pectate lyases (PLs) (Voxeur et al. 2019). PGs are critical for tomato cell wall maceration during *B. cinerea* infection. *Bc*PG1 is highly expressed at later stages of infection and is crucial for pathogen virulence (Have et al. 1998). Mutants affected in two PMEs did not reveal virulence defects on tomato and grapevine leaves. Moreover, loss of PME function did not affect the degradation of methyl esterified pectin, suggesting that *B. cinerea* does not require previous pectin demethylation for its degradation (Kars et al. 2005). *Botrytis cinerea* degradation of *Arabidopsis thaliana* (hereafter Arabidopsis) cell walls results in modified oligogalacturonides (OGs). Using a sensitive analytical profiling method, Voxeur et al. (2019) revealed that during Arabidopsis infection by *B. cinerea*, ~80% of the OGs were generated by fungal PNLs acting on methylesterified homogalacturonan, while PGs targeting unmethylesterified regions contribute only the remaining 20%. PNL activity primarily yields acetyl- and methyl-esterified OGs of DP4-5, whereas PG activity produces shorter dimers and trimers (Voxeur et al. 2019). *BcPNL1*, the most highly expressed *PNL* in *B. cinerea* during Arabidopsis infection, contributes to virulence when plants are grown with high nitrogen concentrations. The products of *Bc*PNL1 activity suppress JA-mediated defences (Davière et al. 2025).

*Fusarium oxysporum* growth in the presence of polygalacturonic acid and pectin induces the expression of two genes encoding GH28 endo-PGs (*PG1* and *PGx4)* and two GH28 exo-PGs (*PG5* and *PGx6*). The double mutants of *PG1* and *PGx6* severely reduced *F. oxysporum* virulence on tomato, suggesting that synergistic activity among endo- and exo-acting pectinolytic enzymes is critical for pathogen full virulence (Bravo Ruiz et al. 2016). In *M. oryzae*, deletion of the most highly expressed PL gene (*MoPL1*) led to a reduction of virulence, suggesting the implication of these pectin degrading enzymes in pathogenicity (Wegner et al. 2022).

The complexity of its lateral chains challenges pectin degradation. Thus, besides pectinases, pathogens secrete other CWMEs to decompose pectin. For example, *B. cinerea* secretes the GH43 endo-arabinase *Bc*Ara1 to hydrolyse the arabinan long chains that are mostly associated with rhamnogalacturonan I. *BcAra1* is upregulated during infection and growth on arabinan-rich substrates. *Bc*Ara1 hydrolises arabinan, enabling its use as a carbon source. Interestingly, *Bc*Ara1 acts as a host-specific virulence factor, contributing to virulence in Arabidopsis but not in *Nicotiana benthamiana* (Nafisi et al. 2014).

### Lignin degradation

The highly variable composition of lignin suggests that pathogens must develop highly specialized degradation strategies, including the release of lignin peroxidases, laccases, manganese-dependent peroxidases, and versatile peroxidases, classified in AA1 and AA2 families and specialized in the oxidative cleavage of phenolic and nonphenolic sites in lignin (Hatakka 1994). Wood-rotting fungi are the primary lignin degraders and are capable of using it as a carbon source (Janusz et al. 2017). Lignin-degrading enzymes are required for epiphytic growth and for penetration of the lignified bark of vascular plants. *Cryphonectria parasitica*, an ascomycota causing chestnut blight, exemplifies this strategy. During infection, it grows epiphytically in continuous contact with the bark of the chestnut tree. Chestnut lignin is rich in the polyphenol tannic acid. Polyphenols induce the expression of fungal laccases. Specifically, the *C. parasitica* laccase Lac3, an AA1 enzyme, contributes to virulence (Chung et al. 2008). AA1 family laccases are multicopper oxidases that oxidize recalcitrant phenolic compounds and are involved in lignin degradation. Although it is suggested that this activity may be required for the virulence of filamentous pathogens, the capacity of laccases to degrade lignin has only been directly correlated to the virulence of *C. parasitica*.

Interestingly, lignin is also an appreciated substrate during the saprotrophic growth of fungi. Ectomycorrhizal fungi secrete CWMEs to degrade lignocellulose (Kohler et al. 2015). This set of enzymes enables an additional nutrient source for some mycorrhizal fungi (Lindahl and Tunlid 2015). Moreover, secreted lignocellulosic enzymes enable saprotrophs to extract nutrients from plant material, which could have potentially facilitated an evolutionary transition into certain plant pathogens (Gordon and Leveau 2010).

Lignin is deposited by the host as an immune response to prevent infection. Wheat root colonization by the endophytic fungus *Gaeumannomyces hyphopodioides* and the pathogenic fungus *Gaeumannomyces tritici* results in an early activation of lignin biosynthetic genes and cell wall lignification (Chancellor et al. 2024). Furthermore, lignin depositions in secondary plant cell walls are induced as an immune response in Arabidopsis against avirulent strains of the pathogenic bacteria *Pseudomonas syringae*. This lignin deposition spatially restricts the mobility of the pathogen and constrains the infection zone (Lee et al. 2019). This exemplifies a stage of the evolutionary arms race in which the plant responds to pathogen effectors, specifically modifying the plant cell wall composition. However, the conflict continues, as pathogens may evolve mechanisms to degrade this physical barrier. For example, the weaponry of AA1 laccases and AA2 peroxidases that target lignin (Hatakka 1994) might enable avirulent strains to overcome lignin depositions. This idea of pathogen counter-responses to plant cell wall fortifications can be extended to other inducible physical defences. One example is callose depositions. Callose is deposited at penetration sites of biotrophic and necrotrophic pathogens. Potentially, callose might be directly targeted by pathogen exoglucanases to breach this physical barrier. In fact, several pathogen effectors target immune signalling pathways to reduce the deposition of callose (Bhandari et al. 2025).

### Specialization of CWMEs repertoire in filamentous pathogens

The specialization of filamentous pathogens into distinct lifestyles and host plants is associated with the acquisition of specific repertoires of CWMEs. Biotrophic pathogens typically encode fewer CWMEs than hemibiotrophs and necrotrophs, with necrotrophs exhibiting the largest amount. In addition to differences in abundance, the composition of CWMEs also varies according to the lifestyle. Necrotrophs and hemibiotrophs encode higher numbers of AA9 cellulases and GH78, PL1 or PL3 pectinases than biotrophs and nonpathogenic fungi (Zhao et al. 2013). Indeed, CWME repertoires have been used to classify pathogens according to their trophic phenotypes, instead of considering the pathogen’s lifestyle (Hane et al. 2020). Specialization of CWMEs to host species is exemplified by the differences in pectin-targeting enzymes between pathogens of monocot or dicot plants. In particular, dicot pathogens encode higher numbers of pectinases from the GH28, GH88, and GH105 families than pathogens infecting grasses (King et al. 2011). Although the adaptation of CWME repertoires is described here in the context of plant pathogens, other plant-associated microorganisms are also adapted to the available nutrients, including those derived from plant cell walls. Remarkably, evolutionary-distant fungi colonizing the same plant host, Arabidopsis, harbour a common set of CWMEs likely involved in nutrient acquisition from plants and root colonization (Mesny et al. 2021). Thus, the capacity to degrade specific cell wall components is critical for adaptation of plant microbiota members to their host and determines host-compatibility and pathogen evolution (Mesny et al. 2023, Raja-Kumar et al. 2025).

## Plant defence: overcoming cell wall damage

**Figure 2:**
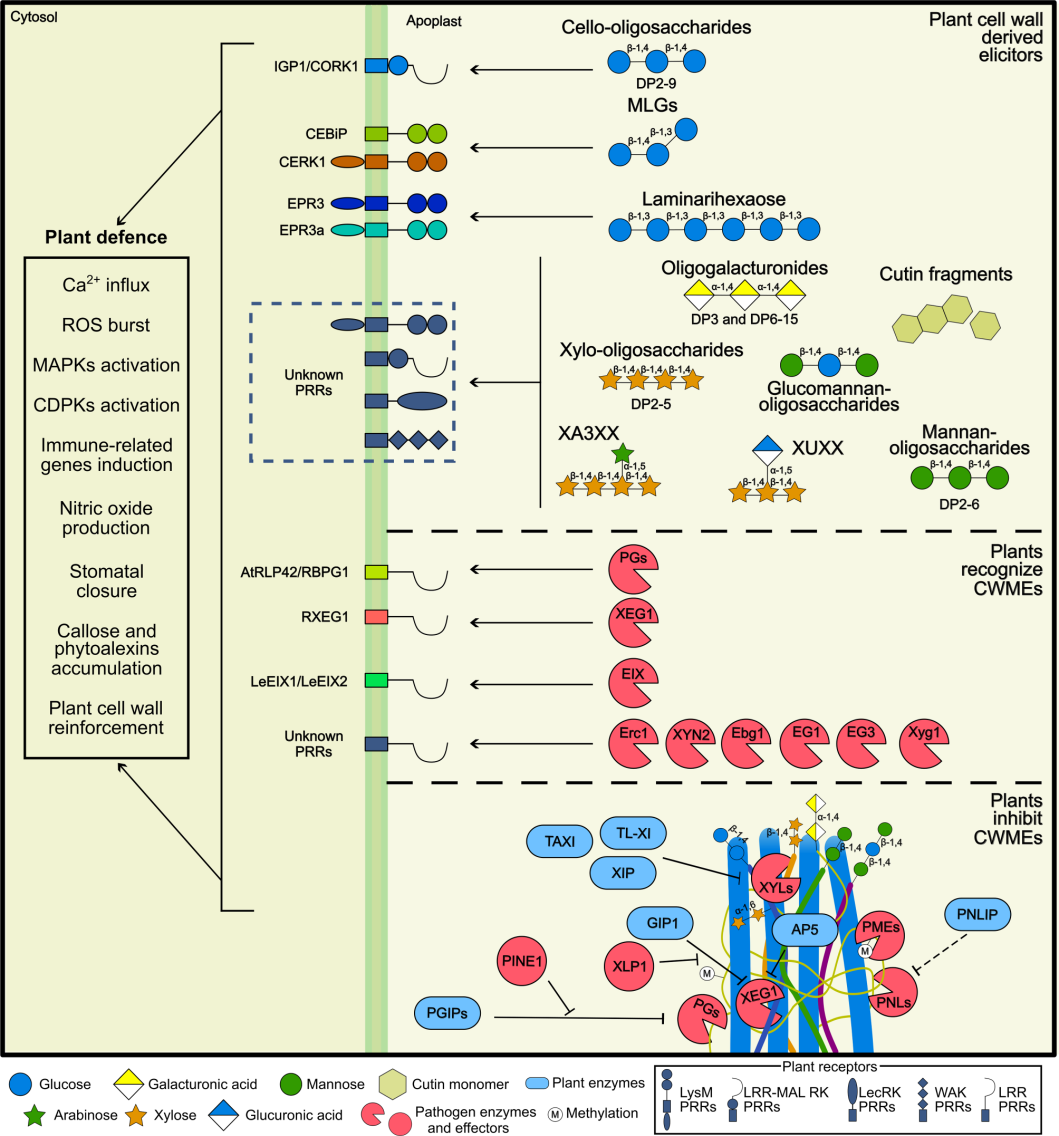
Plant defence. Degradation of plant cell walls releases oligosaccharides that are recognized by plant receptors and trigger an immune response. Only a few *bona fide* receptors have been identified: the pattern recognition receptors for cello-oligosaccharides, MLGs, and laminarinohexaose. Plant cells also harbour receptors that recognize cell wall-modifying enzymes. Finally, plant defence includes the secretion of plant proteins that inhibit the activity of pathogen CWMEs.

Cell walls are dynamic structures that act as defence barriers. Using a multilayered surveillance system, plants monitor cell wall integrity by detecting changes in the structure and composition of cell walls and perceiving released oligosaccharides. The recognition of pathogen CWMEs, reinforcement of plant cell walls, and biochemical inhibition of pathogen enzymes are all part of the host defensive strategy to prevent pathogen progression (Fig. 2) (Molina et al. 2024a, Sun et al. 2022, 2025b, Juge 2006, Bacete et al. 2018, Xia et al. 2020, Wolf 2022, Bhandari et al. 2025). As a consequence, hosts exploit the pathogens’ need to breach host walls to initiate a robust and specific immune response.

### Cell wall degradation products trigger host immunity

Despite the critical role of CWMEs during plant infection, their degradation products can have detrimental effects on pathogens, as they are potentially recognized by the host as DAMPs, triggering immune responses that restrict colonization. These DAMPs differ in nature depending on the cell wall component from which they originate, and include degradation products of xylans, xyloglucans, arabinoxylans, glucuronoxylans, mannans, glucomannans, callose, MLGs, cutin, pectins, and cellulose. Although DAMPs have been extensively studied and are well-established inducers of plant immunity (Molina et al. 2024a, Sun et al. 2025b, Bacete et al. 2018, Bacete and Hamann 2020, De Lorenzo and Cervone 2022, Wolf 2022), to the best of our knowledge, to date, only pectin-derived OGs, MLGs, and cellulose-derived oligosaccharides have been demonstrated to be released during plant infection by fungal pathogens (Table 1) (Voxeur et al. 2019, Yang et al. 2021, Gámez-Arjona et al. 2022, Rebaque et al. 2025, Davière et al. 2025).

**Table 1:** Characterized fungal cell wall-modifying enzymes releasing DAMPs during infection.

CWME	CAZy family	Pathogen	Host	DAMP	References
*Mo*Cel12A/B	GH12	*M. oryzae*	*O. sativa*	MLG43, MLG443	Yang et al. (2021)
*Zt*GH45	GH45	*Z. tritici*	*T. aestivum*	MLG43	Rebaque et al. (2025)
*Bc*PG1	GH28	*B. cinerea*	*A. thaliana*	OGs	Voxeur et al. (2019)
*Bc*PNL1	PL1	*B. cinerea*	*A. thaliana*	OGs	Voxeur et al. (2019), Davière et al. (2025)
*Bc*PME1/2	CE8	*B. cinerea*	*A. thaliana*	OGs	Voxeur et al. (2019)

OGs are linear oligosaccharides of α-1,4-d-galacturonic acid released from homogalacturonan by pathogen-secreted CWMEs (Hahn et al. 1981, Nothnagel et al. 1983, Cervone et al. 1989, Benedetti et al. 2015, Voxeur et al. 2019). Both short (DP 3) and long (DP 6–15) unmethylesterified OGs trigger a cascade of defence responses in several plant species, including soybean, Arabidopsis, and tobacco, and protect against pathogen attack (Aziz et al. 2004, Ferrari et al. 2007, Galletti et al. 2008, 2011, Benedetti et al. 2015, Davidsson et al. 2017). These resistance responses typically include Ca^2+^ influx, reactive oxygen species (ROS) burst, activation of MAPKs and CDPKs, induction of immune-related genes, nitric oxide production, and increased accumulation of callose and phytoalexins (Davis et al. 1986, Mathieu et al. 1991, Bellincampi et al. 2000, Ferrari et al. 2007, Denoux et al. 2008, Galletti et al. 2008, 2011, Rasul et al. 2012, Davidsson et al. 2017). Wall-associated kinases (WAKs) are protein kinases associated with plant cell walls (He et al. 1996, Brutus et al. 2010). The extracellular domain of WAKs binds demethylated pectin (Wagner and Kohorn 2001, Decreux and Messiaen 2005, Decreux et al. 2006). However, the primary role of WAKs as *bona fide* OG receptors has recently been challenged, as disruption of all Arabidopsis WAK homologues does not impair OG-mediated signalling (Herold et al. 2025), suggesting the involvement of other unknown PRRs. Although ectopic application demonstrates the immunogenic potential of OGs, their impact on plant–pathogen interactions ultimately depends on their release from the plant cell wall during infection (Benedetti et al. 2015). The OGs released *in planta* during Arabidopsis infection with *B. cinerea* are highly complex, where most of the accumulated OGs were acetyl- and methylesterified (Voxeur et al. 2019). Similarly, the infection of Arabidopsis roots with *F. oxysporum* released OGs with oxidized or acetylated groups (Gámez-Arjona et al. 2022). Thus, the *in planta* identified OGs are distinct from those shown to have elicitor capacity (Davidsson et al. 2017, Voxeur et al. 2019, Gámez-Arjona et al. 2022). The profile of OGs released during *in planta* infection might reflect the coevolution of plants and pathogens, in which pathogens produce OGs with low elicitor capacity, and plants modify them to induce an immune response.

Cellulose-derived oligosaccharides are also recognized by the host as DAMPs. β-1,4 oligosaccharides with DP ranging from two to nine glucose molecules activate defence responses, including ROS production, MAPK activation, Ca^2+^ influx, and induction of defence-related genes in plants such as Arabidopsis, tomato, and lettuce (Aziz et al. 2007, de Azevedo Souza et al. 2017, Johnson et al. 2018, Locci et al. 2019, Tseng et al. 2022, He et al. 2023, Martín‐Dacal et al. 2023). In Arabidopsis, three members of the leucine-rich repeat-malectin receptor kinase (LRR-MAL RK) family mediate cello-oligosaccharide perception, with Impaired in Glycans Perception 1 (IGP1/CORK1) acting as the receptor (Tseng et al. 2022, Martín-Dacal et al. 2023). The ectodomain of IGP1/CORK1 binds directly to cello-oligosaccharides with a DP ≥ 3. Structural analyses demonstrated that the LRR domain of IGP1 forms a highly specific pocket that binds cello-oligosaccharides through three aromatic residues (Sun et al. 2025a, Jiménez-Sandoval et al. 2025). Remarkably, the malectin domain acts as a redox sensor that reduces IGP1 affinity to cello-oligosaccharides, likely to prevent overactivation of the immune response and maintain cellular homeostasis (Sun et al. 2025a).

MLG-derived oligosaccharides are not plant-exclusive and are also structural components of oomycete, fungal, bacterial, and algal cell walls (Lee and Hollingsworth 1997, Fontaine et al. 2000, Pettolino et al. 2009, Sørensen et al. 2011, Pérez-Mendoza et al. 2015, Samar et al. 2015, Salmeán et al. 2017, Chang et al. 2023). MLGs trigger resistance in both monocots and dicots, activating distinct sets of immune responses, including ROS burst, phosphorylation of MPKs, induction of defence-related genes, stomatal closure, and cytoplasmic Ca^2+^ elevations. Consequently, exogenous pretreatment with these oligosaccharides protects crops against bacterial and fungal diseases (Barghahn et al. 2021, Rebaque et al. 2021, Yang et al. 2021, Martín‐Dacal et al. 2023, 2025). MLG perception in plants involves multicomponent receptor complexes. In Arabidopsis, recognition of MLGs requires the LysM-containing receptors AtCERK1, LYK4, and LYK5 and the IGP1/CORK1, IGP3, and IGP4 LRR-MAL RKs (Rebaque et al. 2021, Martín‐Dacal et al. 2023). In rice, MLGs are recognized by OsCERK1, inducing its dimerization with OsCEBiP (Yang et al. 2021). In addition, MLGs released during rice-brown planthopper interactions are recognized by OsLecRK1, highlighting the possible involvement of lectin-type receptors in MLG sensing and immune activation (Dai et al. 2023). *Mo*Cel12A/B and *Zt*GH45 from *M. oryzae* and *Z. tritici*, respectively, release immunogenic MLG-derived oligosaccharides during infection (Yang et al. 2021, Rebaque et al. 2025). Interestingly, 3^1^-β-d-Cellobiosyl-glucose (MLG43) released during wheat infection by *Z. tritici* induces stomatal closure (Rebaque et al. 2025), one of the most significant entry points for various pathogens (Melotto et al. 2024).

Beyond its structural role as a physical barrier, callose serves as a source of elicitors. Based on the innate capacity to perceive endogenous glucans, plants also recognize structurally analogous microbial β-1,3-linked glucan-derived oligosaccharides, such as laminarihexaose and laminarin oligosaccharides, which are included in this review due to their homology with plant cell wall components. Laminarin is a β-1,3-glucan containing one or more glucose units linked via a β-1,6 bond as side branches. While CERK1 seems to act as a coreceptor for linear β-1,3-glucans in Arabidopsis, their recognition is CERK1-independent in rice and *N. benthamiana* (Mélida et al. 2018, Wanke et al. 2020). In *Lotus japonicus*, the receptor kinases EPR3 and EPR3a bind laminarin, mediating perception of this oligosaccharide during symbiotic interactions (Kelly et al. 2023). The defence responses triggered by these oligosaccharides in both monocots and dicots include an increase in cytosolic Ca^2+^ levels, ROS production, MAPK activation, and induction of defence-related gene expression (Klarzynski et al. 2000, Aziz et al. 2003, 2007, Wawra et al. 2016, Mélida et al. 2018, Wanke et al. 2020, 2023, Kelly et al. 2023).

Xylose-rich hemicelluloses are also a source of oligosaccharides that are immunogenic. Linear β-1,4-d-xylo-oligosaccharides with DP ranging from 2 to 5 can activate a wide range of defence responses in Arabidopsis, tomato, and wheat. These may include increased calcium influx, ROS production, MAPK phosphorylation, callose deposition, and the induction of defence-related genes (Dewangan et al. 2023, Pring et al. 2023, Fernández-Calvo et al. 2024). In *Vitis vinifera* and Arabidopsis, a mix of xyloglucan-derived oligosaccharides activates a broad range of defence responses, resulting in increased resistance to *B. cinerea* and *Hyaloperonospora arabidopsidis* (Claverie et al. 2018). The pentasaccharide XA3XX (3^3^-α-l-arabinofuranosyl-xylotetraose) with a branched-arabinose monosaccharide is highly immunogenic in Arabidopsis, tomato, and pepper, protecting tomato and pepper plants against *P. syringae* and *Sclerotinia sclerotiorum*, respectively (Mélida et al. 2020, Fernández-Calvo et al. 2024). Interestingly, in Arabidopsis, the recognition of both xylotetraose and XA3XX also requires the LRR-malectin receptor kinases IGP1/CORK1, IGP3, and IGP4, highlighting the relevance of these receptors in glycan-mediated immunity (Fernández-Calvo et al. 2024). Other xylans, like those derived from glucuroxylans, such as XUXX (2^3^-(4-O-methyl-α-d-glucuronyl)-xylotetraose), also induce a resistance response in Arabidopsis (Fernández-Calvo et al. 2024).

Mannan-derived oligosaccharides (MOS) with a DP of 2–6 also induce resistance in rice and tobacco. MOS leads to ROS and intracellular calcium ion accumulation, and to cell death and stomatal closure. Interestingly, these oligosaccharides enhanced the resistance of *Oryza sativa* and *N. benthamiana* against *Xanthomonas oryzae* and *Phytophthora nicotianae*, respectively (Zang et al. 2019). In *N. benthamiana*, the perception of glucomannan-derived oligosaccharides triggers H_2_O_2_ and callose accumulation, activates the salicylic acid and jasmonic acid/ethylene pathways, and leads to a marked reduction in lesions caused by *P. nicotianae* (Rajib et al. 2024).

Beyond their structural function, cuticle-derived components also serve as a source for immune elicitors. Cutin monomers and oligomers trigger defence responses in plants (Schweizer et al. 1996, Kauss et al. 1999, Kim et al. 2008, Park et al. 2008), and induce calcium influx, ROS production, MAPK activation, and PTI-associated transcriptional reprogramming (Moreira et al. 2024).

The wide diversity of cell wall-derived oligosaccharides recognized by plants reflects a sophisticated coevolutionary arms race. Since pathogens depend on the secretion of CWMEs to breach the plant cell wall and access nutrients, the plant has turned this necessity into a vulnerability by evolving a robust machinery that supervises wall integrity. This recognition system exerts selective pressures on pathogens, which evolved strategies to mask their enzymatic activity, such as hydrolysis, modification, or sequestration of released DAMPs, or the fine-tuning of CWME expression. The plant immune system has evolved to make the cell wall a dynamic defence system, where the chemical nature and concentration of the released DAMPs determine the outcome of the interaction.

### Plant recognition of CWMEs

Plants additionally recognize specific CWMEs, irrespective of their catalytic activity or the elicitors they generate (Table 2). This recognition elicits immune responses that complement those activated by cell wall-derived DAMPs. One of the earliest examples of CWMEs being recognized by plants came from endo-β-1,4-xylanases secreted by *Trichoderma viride* and *T. reesei* (Fuchs et al. 1989, Bailey et al. 1990, Enkerli et al. 1999). The GH11 EIX and XYN2 trigger ethylene production and necrosis in tobacco and tomato (Bailey et al. 1990, 1991, Enkerli et al. 1999, Ron et al. 2000), and this elicitor activity is independent of their catalytic function, as mutated versions of both enzymes with reduced β-1,4-xylanase activity still triggered host immunity (Sharon et al. 1993, Enkerli et al. 1999, Furman-Matarasso et al. 1999, Rotblat et al. 2002). Following these initial discoveries, several additional CWMEs with similar elicitor properties have been identified across diverse fungal pathogens. The *P. sojae* GH12 *Ps*XEG1 is recognized by soybean and solanaceous plants, independently of its enzymatic activity, triggering an immune response (Ma et al. 2015b). Its receptor in *N. benthamiana*, the LRR protein RXEG1, forms a complex with BRI1-associated receptor kinase 1 (BAK1) upon ligand binding (Sun et al. 2022). Other GH12 enzymes from *B. cinerea, V. dahliae*, and *F. oxysporum* are also recognized by plants, independently of their enzymatic activity (Zhang et al. 2021b, Zhu et al. 2017, Gui et al. 2017). Beyond the GH12 family, the capacity of CWMEs to elicit immunity extends to other classes of CWMEs, such as ERC1, a GH51 glucanase from *U. maydis* that hydrolyses β-1,3 linkages (Ökmen et al. 2022); EG1, a GH45 endoglucanase secreted by *Rhizoctonia solani* (Ma et al. 2015a, Guo et al. 2022); and Ebg1, a GH17 exo-β-1,3-glucanase from *M. oryzae* (Liu et al. 2023).

**Table 2:** Cell wall-modifying enzymes from fungal pathogens recognized by plant hosts.

CWME	CAZy family	Pathogen	Plant species tested	References
EIX	GH11	*T. viride*	*S. lycopersicum* and *N. tabacum*	Bailey et al. (1990), Ron et al. (2000)
XYN2	GH11	*T. reesei*	*S. lycopersicum* and *N. tabacum*	Enkerli et al. (1999)
*Ps*XEG1	GH12	*P. sojae*	*G. max* and solanaceous plants	Ma et al. (2015b)
*Bc*Xyg1	GH12	*B. cinerea*	*N. benthamiana, P. vulgaris*, and *S. lycopersicum*	Zhu et al. (2017)
*Vd*EG1/*Vd*EG3	GH12	*V. dahliae*	*N. benthamiana*	Gui et al. (2017).
*Fo*EG1	GH12	*F. oxysporum*	*N. benthamiana, N. tabacum, S. lycopersicum*, and *G. hirsutum*	Zhang et al. (2021b)
Ebg1	GH17	*M. oryzae*	*O. sativa*	Liu et al. (2023)
PGs	GH28	*B. cinerea/A. niger*	*A. thaliana*	Zhang et al. (2014, 2021a)
EG1	GH45	*R. solani*	*A. thaliana, N. benthamiana*, and *Z. mays*	Ma et al. (2015a), Guo et al. (2022)
Erc1	GH51	*U. maydis*	*Z. mays* and *H. vulgare*	Ökmen et al. (2022)

CWME recognition is generally considered a broadly conserved trait at the species level. However, some exceptions have been described. PGs from *B. cinerea* and *Aspergillus niger*, classified within the GH28 family, are recognized by the Arabidopsis leucine-rich repeat receptor-like protein *At*RLP42/RBPG1. However, not all Arabidopsis accessions respond to *Bc*PGs. While some accessions induce strong necrosis upon protein infiltration, others show little or no response (Zhang et al. 2014, 2021a). Similarly, recognition of EIX is cultivar-specific in tobacco and tomato (Bailey et al. 1993, Avni et al. 1994, Ron et al. 2000), and is mediated by the LRR receptor-like proteins LeEIX2 and LeEIX1 in a BAK1-dependent manner (Ron and Avni 2004, Bar et al. 2010). These findings suggest that perception of CWMEs is genotype-specific and therefore not fully conserved within plant species. We hypothesize that, in general, specific recognition of CAZymes occurs at a plant and pathogen genotype-specific level. This proposed genotype-specific recognition suggests a strong selective pressure on the plant to recognize CWMEs and on the pathogen to diversify their CWME sequences to escape from recognition.

### Plants inhibit CWMEs

Plants can also counteract pathogen attack by inhibiting CWMEs. One of the best-studied cases involves PG-inhibiting proteins (PGIPs), described over five decades ago (Albersheim and Anderson 1971), which bind pathogen PGs and suppress their hydrolytic activity. PGIPs are a superfamily of LRR proteins broadly distributed in plants, localized at the plant cell wall, and highly expressed during infection (De Lorenzo et al. 2001, Kalunke et al. 2015). Several studies have shown that PGIPs are key determinants of plant resistance to a wide range of fungal, oomycete, and bacterial pathogens, underscoring their pivotal role in immunity (Ferrari et al. 2003, 2006, Kalunke et al. 2015). Remarkably, PGIPs exhibit high specificity, recognizing only particular PG isoforms. This reflects a continuous coevolutionary arms race where plants might evolve to target specific PGs, while pathogens might simultaneously evade this inhibitory activity by altering the PG sequence (Leckie et al. 1999, Wang et al. 2021). Additionally, some pathogens have evolved alternative strategies to prevent PGIP-mediated immunity. The secreted small effector from *S. sclerotiorum* PGIP-INactivating Effector 1 (*Ss*PINE1) binds directly to *At*PGIP1 and impairs its inhibitory function, thereby facilitating PG-mediated pectin hydrolysis (Wei et al. 2022). Deletion of *Ss*PINE1 reduces virulence, whereas its ectopic expression in Arabidopsis increases susceptibility against *S. sclerotiorum*, highlighting the contribution of this effector in virulence. *Ss*PINE1 is broadly distributed, suggesting that neutralizing host PGIPs may be a conserved strategy for pathogens to overcome PGIP-mediated resistance and successfully colonize their host (Wei et al. 2022). In addition to inhibiting, plants hijack pathogen PGs to reinforce host immunity (Xiao et al. 2024). The stable interaction between *Phaseolus vulgaris* PGIP2 (*Pv*PGIP2) and *Fusarium phyllophilum* PG (*Fp*PG) does not block the active site of the enzyme, as described for other proteins inhibiting CWMEs (Payan et al. 2004, Sansen et al. 2004, Di Matteo et al. 2005, Sun et al. 2022). Instead, the *Pv*PGIP2–*Fp*PG complex forms a novel substrate-binding site with enhanced affinity for polygalacturonic acid and an altered cleavage pattern. This complex favours the generation of long-chain immunogenic OGs while simultaneously diminishing the production of short fragments. The OGs released by the *Pv*PGIP2–*Fp*PG complex trigger robust immune responses in both soybean and Arabidopsis (Xiao et al. 2024). *Pv*PGIP2 represents a specialized strategy in which plants sequester pathogen virulence factors to trigger defence signalling.

Beyond PGIPs, plants produce other protein inhibitors of pathogen CWMEs. These include pectin methylesterase inhibitors (PMEIs) and pectin lyase inhibitors (PNLIs). PMEIs form a large family of small secreted proteins that inhibit PMEs, preventing homogalacturonan demethylesterification and thereby maintaining cell wall rigidity and limiting pathogen access (Wolf et al. 2009, Wormit and Usadel 2018). Evidence from several plant pathosystems supports the contribution of PMEIs to plant resistance. For instance, Arabidopsis *At*PMEI1, 2 10, 11, and 12 contribute to resistance against *B. cinerea* (Lionetti et al. 2007, 2017), cotton *Gh*PMEI3 is involved in resistance to *V. dahliae* (Liu et al. 2018), *AcPMEI* from kiwi confers resistance to *Bipolaris sorokiniana* and *Fusarium graminearum* in durum wheat (Volpi et al. 2011), and *HvPEI4* mitigates *Rhynchosporium commune* infection in barley (Marzin et al. 2016). However, no experimental evidence has shown that plant PMEIs directly interact with or inhibit microbial PMEs. PNLIs are less characterized than PGIPs but also play a role in plant immunity. The sugar beet pectin lyase inhibitor protein (PNLIP) inhibits fungal PNLs from *R. solani* and *Phoma betae* (Bugbee 1993). These findings highlight that the production of PMEIs and PNLIs represents a potential plant strategy to maintain cell wall integrity, hindering pathogen enzymatic attack and subsequent colonization.

Plants have also evolved specialized inhibitors targeting hemicellulose-degrading enzymes, particularly fungal endoxylanases. In fact, three types of xylanase inhibitors, namely TAXI (*Triticum aestivum* xylanase inhibitor; Debyser et al. 1999), XIP (xylanase inhibitor protein; McLauchlan et al. 1999), and TL-XI (thaumatin-like xylanase inhibitor; Fierens et al. 2007) are abundant in cereals (Goesaert et al. 2004). They specifically inhibit microbial GH11 and/or GH10 endo-β-1,4-xylanases without affecting endogenous plant xylanases (Gebruers et al. 2001, Flatman et al. 2002, Fierens et al. 2003, Payan et al. 2004, Beliën et al. 2005, Brutus et al. 2005, Furniss et al. 2005, Juge 2006, 2007, Misas-Villamil and van der Hoorn 2008, Pollet et al. 2009). For example, wheat endoxylanase inhibitors from XIP and TAXI families effectively mitigate the activity of GH11 enzymes from *B. cinerea* and *F. graminearum* (Beliën et al. 2005, Brutus et al. 2005, Tundo et al. 2020), and contribute to resistance of durum wheat to *F. graminearum* (Moscetti et al. 2013, 2015, Tundo et al. 2015). Beyond wheat, the pear apoplastic inhibitor *Pb*XIP1 binds to *C. fructicola* xylanase *Cf*Xyn11A, leading to a marked reduction in its enzymatic activity and an enhanced resistance (Han et al. 2025), and the rice XIP-type inhibitors RIXI or OsXIP enhance resistance to *M. oryzae* (Hou et al. 2015, Sun et al. 2018). Overall, these findings reinforce the role of xylanase inhibitors as a strategy to limit fungal xylanase activity during infection.

The relevance of pathogen CWMEs and plant inhibitors is nicely illustrated in the pathosystem *P. sojae*–soybean. The pathogen secretes the GH12 xyloglucanase *Ps*XEG1, a positive regulator of virulence, while soybean counters this attack with the apoplastic glucanase inhibitor protein 1 *Gm*GIP1. This protein binds *Ps*XEG1 with high specificity and blocks its enzymatic activity, promoting resistance to *P. sojae*. To overcome this inhibition, *P. sojae* secretes a paralogous protein of *Ps*XEG1, *Ps*XLP1, which lacks the xylanase catalytic activity but binds *Gm*GIP1 with five-fold higher affinity. By acting as a molecular decoy and sequestering *Gm*GIP1, *Ps*XLP1 plays a critical role in *P. sojae* virulence. *Ps*XLP1 functions as a virulence factor that neutralizes host inhibitors through competitive binding, acting as a molecular decoy (Ma et al. 2017).

Finally, the degradation of pathogen-secreted CWMEs represents an alternative strategy to prevent infection. In soybean, the aspartic protease *Gm*AP5 specifically targets and degrades the *Ps*XEG1 effector, thereby mitigating pathogen progression. However, N-glycosylation of *Ps*XEG1 at residues N174 and N190 provides a structural shield that protects the effector from both *Gm*AP5-mediated proteolysis and *Gm*GIP1-mediated inhibition during infection (Xia et al. 2020). Together, these findings reveal a multifaceted coevolutionary arms race within the apoplast. This competition involves distinct strategies, ranging from the direct inhibition of enzymatic activity to the proteolytic degradation of CWMEs. In this context, host responses can prevent cell wall damage or even overcome pathogen virulence factors, while pathogens counterattack through structural modifications of their CWMEs or the deployment of molecular decoys to bypass these host defences.

### Plant cell wall fortification

The cell wall structure and composition are highly dynamic and are modified depending on the environmental conditions (Rui and Dinneny 2020, Bhandari et al. 2025). In fact, plants activate the expression of several CWME-encoding genes as a key component of the immune response. For example, resistant tomato plants activate the expression of genes involved in the synthesis of lignin and suberin upon infection by the bacteria *Ralstonia solanacearum* (Kashyap et al. 2022). Potato cells in intimate contact with the oomycete *Phytophthora infestans* suffered local transcriptional remodelling characterized by the upregulation of genes associated with cell wall modifications (Li et al. 2026). This tightly regulated transcriptomic response highlights the critical role of controlled and localized plant cell wall reinforcements as a defence strategy against pathogens. In fact, certain depositions at the cell walls during infection are key to halting the progression of the infection. For instance, callose, a β-1,3-glucan, is deposited as an early defence response at the penetration sites of several pathogens, including *Blumeria graminis* f. sp. *hordei*, to mitigate pathogen invasion (DebRoy et al. 2004, Chowdhury et al. 2014, 2016). Callose interacts with cellulose and forms a mesh that constitutes a physical barrier to hinder pathogen penetration. In addition, callose is associated with antimicrobials and hydrogen peroxide in the so-called papillae that are a strong barrier against pathogen penetration (Voigt 2014, Munzert and Engelsdorf 2025). Lignin is an additional cell wall component that is deposited during plant infection (Miedes et al. 2014). Larger levels of lignin are accumulated in Arabidopsis in response to the infection by avirulent strains of the bacterial pathogen *P. syringae* compared to virulent strains. This localized lignin deposition restricts the movement of the avirulent strain and prevents its spread to adjacent cells of the leaf, forming a very robust physical barrier (Lee et al. 2019). Lignin-based cell wall reinforcements also occur in resistant tomato plants to contain the movement of bacteria. Resistant tomato plants form a ligno-cellulose barrier at the vasculature upon infection by *R. solanacearum*. These cell wall depositions prevent bacterial degradation of the plant cell wall and its progression in the plant (Kashyap et al. 2022). Further cell wall remodelling occurs upon infection with *P. syringae* in Arabidopsis. As part of the plant immune response, Arabidopsis triggers the deposition of arabinose and xylose in cell walls of epidermal and mesophyll cells (Kim et al. 2023). Pectins are also modified to increase stiffness of the plant cell wall. Demethylation of homogalacturonan is promoted in Arabidopsis upon infection by *B. cinerea*, most probably to facilitate pectin cross-linking and prevent pathogen penetration (Coculo et al. 2023). These cell wall modifications might have distinct contributions to plant immunity, such as constituting a physical barrier, acting as signals to amplify the immune response, or preventing the action of pathogen CWMEs (Bhandari et al. 2025). Although the contribution of cell wall modifications to defence is still not well understood, they may represent a key strategy for preventing pathogen attack.

## Pathogen counterattack: evading host immunity

**Figure 3:**
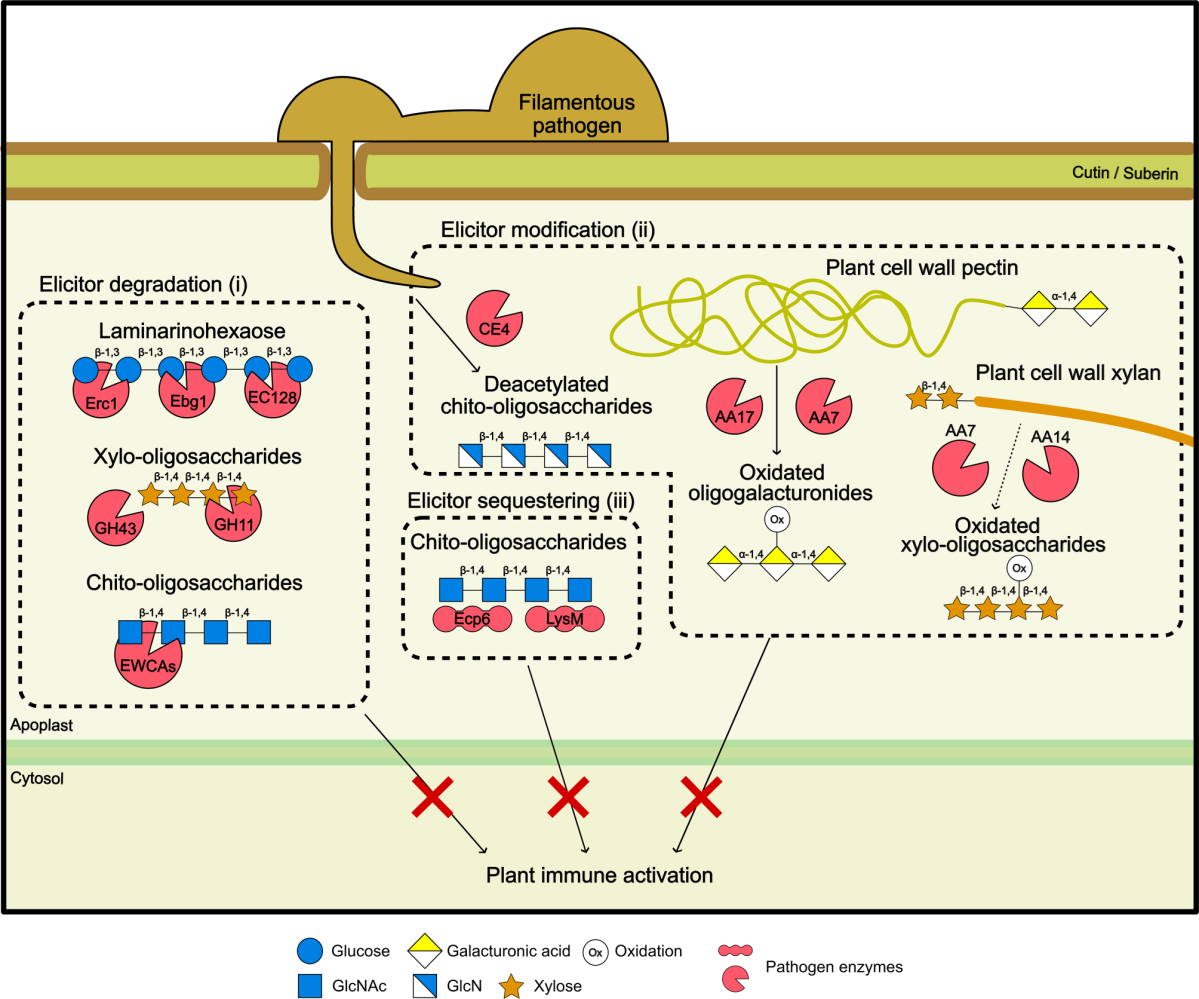
Pathogen counterattack. Filamentous pathogens have evolved to suppress the plant immune response. Pathogens secrete cell wall-modifying enzymes to (i) degrade; (ii) modify, and (iii) sequester cell wall-derived elicitors to prevent their recognition by plant receptors.

Host recognition of cell wall-degradation products poses an evolutionary pressure on the pathogen. Pathogens have evolved strategies to circumvent this immune response. A common mechanism involves reducing the abundance or availability of recognized immunogenic cell wall-derived oligosaccharides (Sun et al. 2025b). To this end, pathogens can degrade, modify, or sequester cell wall-derived elicitors (Fig. 3) or tightly regulate the activity of elicitor-releasing CWMEs.

### Degradation of released elicitors

An efficient strategy to escape from recognition involves the degradation

of cell wall-derived elicitors. *Serendipita indica* coexpresses a cocktail of enzymes that target xylan. The acetylxylan esterase *Si*AXE and the endoxylanase *Si*GH11 release xylooligosaccharides, which are recognized by the host, and may thereby hamper fungal colonization. Their coordinated activity with *S. indica* exoxylanases (most probably from GH43 family) leads to the degradation of the generated elicitors, contributing to the colonization of barley roots (Brands et al. 2025). Despite the low number of characterized enzymes degrading plant cell wall-derived elicitors, several CAZymes have been shown to degrade pathogen-derived elicitors. Pathogens have developed distinct strategies to reduce the concentration of immunogenic chitin oligosaccharides during plant infections (Kaku et al. 2006, Sánchez-Vallet et al. 2015). *Podosphaera* spp. harbours a highly conserved family of enzymes with chitinase activity, which are released at the penetration sites and degrade chitin oligosaccharides, thus preventing the induction of the immune response (Martínez-Cruz et al. 2021). An additional CAZyme, *Px*LPMO1, which is classified as AA11, is highly expressed in the haustoria of *Podosphaera xanthii* and degrades chitin oligosaccharides and, thereby prevents chitin recognition by the host and enables plant infection (Polonio et al. 2021).

β-glucans are present in both pathogen and host cell walls. Thus, potentially, immunogenic degradation products derived from these polysaccharides might be targeted by the same enzymes. Oligosaccharides derived from β-1,3-glucans, which are present in cell walls from filamentous pathogens and in plant callose depositions, trigger plant defence responses. Several effectors with β-1,3-glucanase activity mediate virulence. *Ustilago maydis* Erc1, a GH51 exo-β-1,3-glucanase, *M. oryzae* Ebg1, a GH17 family protein, and *Colletotrichum graminicola Cg*EC124, a GH128 protein, degrade β-1,3-glucan oligosaccharides to hinder the induction of plant immunity and are required for full virulence (Ökmen et al. 2022, Liu et al. 2023, Gu et al. 2024). Furthermore, a conserved GH64 with putative β-1,3-glucanase activity promotes root infection in two different fungal species (*Plectosphaerella cucumuerina* and *Colletotrichum incanum*) (Raja-Kumar et al. 2025). These studies highlight the pivotal role of the degradation of elicitors as a conserved immune evasion strategy. Although more cases are known for MAMPs, we speculate that similar mechanisms also target plant cell wall-derived oligosaccharides to enable stealth growth of the pathogen.

### Modification of released elicitors

An alternative strategy to reduce the concentration of cell wall derived elicitors is to modify their structure or composition. Oxidation and acetylation of hydroxyl groups, methyl-esterification of carboxyl groups, sulfation, phosphorylation, and conjugation with functional groups of cell wall-derived elicitors might prevent recognition by the host (Muthana et al. 2012). Examples for this scenario come from modifications of fungal cell wall-derived chitin oligosaccharides. These are deacetylated by pathogen deacetylases, such as those of the CE4 family produced by *V. dahliae, P. striiformis* f. sp. *tritici*, and *F. graminearum* to reduce the host immune response and facilitate virulence (Gao et al. 2019, Xu et al. 2020, Hu et al. 2023). Pathogens also modify plant cell wall-derived DAMPs. During the early stages of *P. infestans* infection, a gene encoding a berberine bridge enzyme-like (BBE-like) protein from the AA17 family is strongly induced. This enzyme cleaves pectin, generating oxidized and decarboxylated OGs, which are not recognized by the host, illustrating an alternative strategy to degrade pectins while remaining undetected (Sabbadin et al. 2021). The contribution of BBE-like proteins to virulence has also been demonstrated in *P. sojae* and *P. infestans* AA7 proteins, which oxidize pectin-derived oligosaccharides, such as OGs. These oxidized forms of OGs are less immunogenic and facilitate fungal colonization without being detected (Turella et al. 2025, Welsh et al. 2025). Remarkably, AA7s in *Phytophthora* sp. are coregulated with pectinolytic CAZymes such as GH28, PL3 and AA17, suggesting the coordinated action of pectin degradation and OG inactivation to prevent the induction of the immune response (Welsh et al. 2025). The key role of OG oxidases to mitigate host recognition is probably also critical in other plant pathogens.

Besides pectin, oxidation of other cell wall-derived components has also been shown to be important for virulence. AA7 from the fungus *Myceliophthora thermophila* degrades xylan and produces oxidized xylooligosaccharides (Ferrari et al. 2016). The wood-decay fungus *Pycnoporus coccineus* expresses an AA14 xylan monooxygenase that, together with GH11 xylanases, produces oxidized products (Couturier et al. 2018). Potentially, AA7 and AA14 enzymes can be secreted by filamentous pathogens to release oxidized xylooligosaccharides. Similar to what has been described for oxidized OGs, oxidized xylooligosaccharides may be less immunogenic than non-modified ones. In general, LPMOs can alter plant cell wall-derived elicitors, mainly through oxidation, thereby mitigating their elicitor potential and emerging as key components in the molecular arms race between plants and pathogens to prevent DAMP recognition.

### Sequestering of released elicitors

An additional strategy to prevent the induction of the immune response relies on the sequestering of elicitors. The first characterized fungal effector harbouring a CBM domain was the LysM effector Ecp6 from *Cladosporium fulvum*. Ecp6 contains three tandem lysin motifs (LysM) domains and corresponds to the CBM50 CAZy family (Bolton et al. 2008). Ecp6 binds chitin oligosaccharides with picomolar affinity. This high affinity enables Ecp6 to sequester chitin oligosaccharides and outcompetes the host receptor for chitin binding, and, in this way, it prevents chitin-triggered immunity (de Jonge et al. 2010, Sánchez-Vallet et al. 2013). Alternatively, LysM effectors with only two LysM domains can eliminate chitin oligomers by forming polymeric complexes that precipitate them (Tian et al. 2022). The carbohydrate-binding capacity of CBMs might also be used by pathogens to prevent access by hydrolytic enzymes. The CBM50 *Mg*1LysM from *Z. tritici* prevents the access of chitinases to the fungal cell wall and, in this way, hinders the degradation of the cell wall and the release of oligosaccharides (Sánchez-Vallet et al. 2020). This CBM-based strategy might be used by plant-colonizing microorganisms to sequester other immunogenic oligosaccharides, including those derived from plant cell walls.

### Regulation of fungal CAZymes to prevent early recognition

Pathogens must finely control the timing of CWME expression. Coordinated release of CWMEs is pursued by fungal pathogens to increase virulence. *Botrytis cinerea* can actively modulate host immunity through the release of the PNL *Bc*PNL1 before PGs. PNL activity generates oligosaccharides that induce the expression of host JA repressors, thereby hindering defence responses before further oligosaccharide degradation (Davière et al. 2025). CWME control also aims to prevent early release of immunogenic oligosaccharides and, subsequently, early recognition by the host. For instance, in the *Z. tritici*–wheat interaction, the pathogen restricts the expression of the *ZtGH45* gene during early infection stages to avoid the premature release of MLG-derived DAMPs (Rebaque et al. 2025). By delaying the induction of genes encoding CWMEs, pathogens restrict cell wall degradation to later infection stages when the plant defence is less effective. These strategies of transcriptional fine-tuning represent a critical checkpoint in the evolutionary arms race, where pathogens balance the necessity of breaking the plant cell walls with the risk of activating the host’s innate immune system.

## Concluding remarks

In this review, we discuss the role of plant cell walls in plant–pathogen coevolution. We argue that the cell wall is a dynamic structure that plays a central role in both plant resistance and pathogen survival. To colonize their hosts, pathogens must overcome this barrier, often by deploying a highly specialized arsenal of effectors that degrade cell wall components. These effectors breach the wall and release nutrients that support pathogen growth and colonization. On the contrary, plants have evolved to perceive pathogen-induced modifications of their cell walls, triggering immune responses that restrict pathogen colonization. This interaction imposes strong evolutionary pressure on pathogens, which in turn evolve strategies to modify or degrade cell wall-derived elicitors or fine-tune the CWME expression, thereby evading host recognition. As a result, coevolutionary dynamics take place at the plant cell wall, shaping infection outcomes and the evolution of both plants and pathogens. Although significant progress has been made in understanding the role of plant cell walls in plant–pathogen interactions, critical questions remain unresolved. In particular, we still lack a detailed understanding of how cell wall composition and structure change during resistance responses and which modifications are critical to halt pathogen progression. Addressing this question remains challenging because critical cell wall changes probably occur locally at infection sites, making them difficult to detect experimentally. Furthermore, the DAMPs that are actually produced during plant infection remain largely unidentified. Our understanding of how plants perceive cell wall modifications or damage, and how pathogens counteract plant cell wall-triggered immunity, is still limited. Finally, the CWME repertoire of plant-associated microorganisms is highly specific to the host species and microorganism lifestyle. However, we still do not fully understand what the contribution of CWMEs to pathogenesis, host jumps, and host adaptation is. Because the plant cell wall is central to plant infections, incorporating its dynamic role into the molecular arms race framework will provide a more comprehensive understanding of plant-microorganism coevolution.
